# Supplementary figures and images for: Effector memory CD8 T cell response elicits Hepatitis E Virus genotype 3 pathogenesis in the elderly
Source: PLoS Pathog. 2021 Feb 22;17(2):e1009367. doi: 10.1371/journal.ppat.1009367 (PMC7932504; doi:10.1371/journal.ppat.1009367)

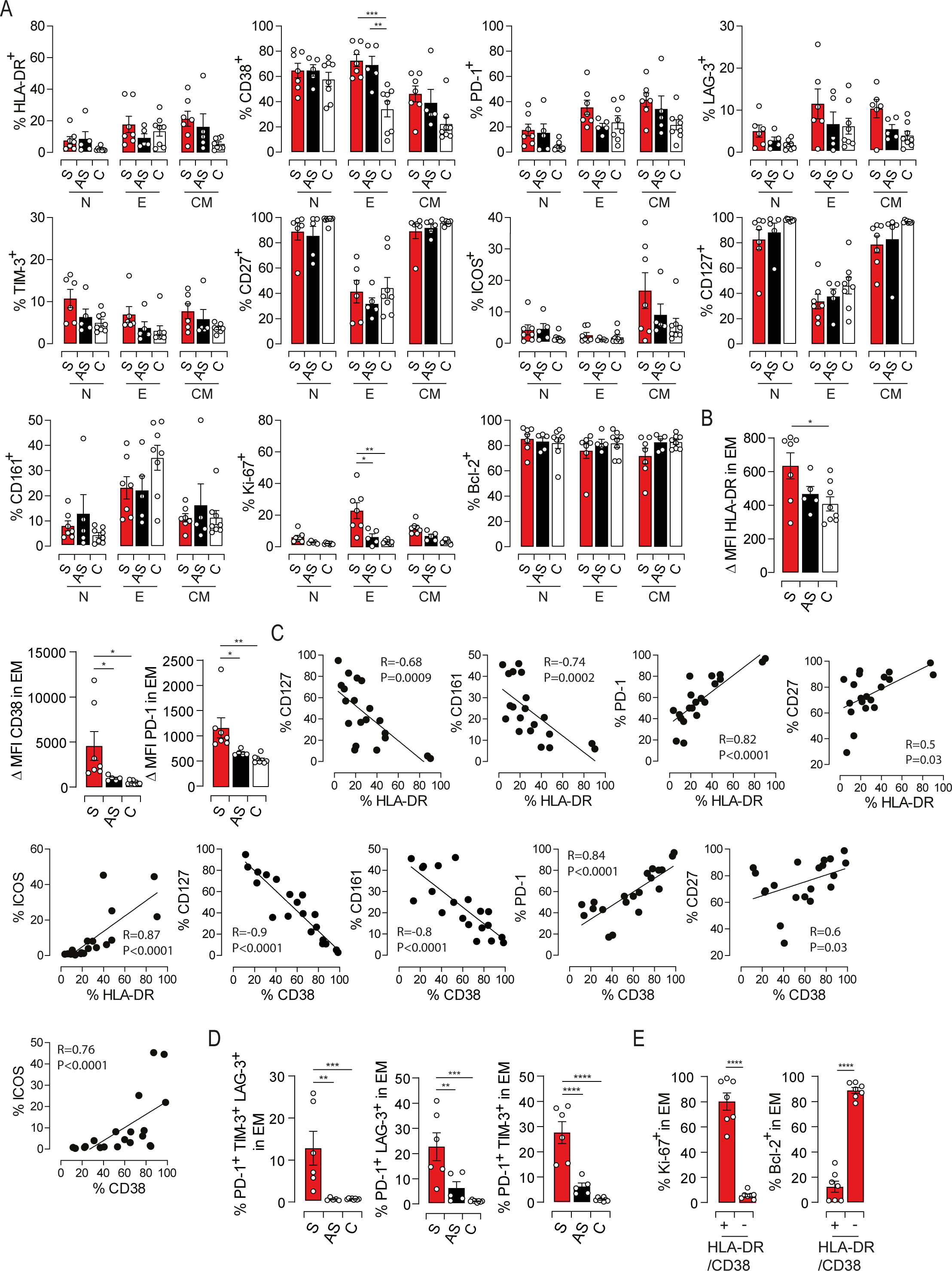

Supplement: S1 Fig — (A) Percentage of cells expressing HLA-DR, CD38, PD-1, TIM-3, LAG-3, CD27, ICOS, CD127, CD161, Ki-67 and Bcl-2 in CD8 T subsets: Naïve (N), Effector (E) and Central Memory (CM). (B) Geometric Mean Fluorescence Intensity of indicated markers in effector memory (EM) CD8 T cells calculated relatively to the negative staining for the concerned marker (ΔMFI). (C) Correlation between the percentage of cells expressing HLA-DR or CD38 and CD127, CD161, PD-1, CD27 or ICOS by Spearman correlation test. (D) Percentage of EM CD8 T cells coexpressing PD-1 with TIM-3, and/or LAG-3. (E) Percentage of cells expressing Ki-67 and Bcl-2 based on the coexpression (+) or absence (-) of HLA-DR/CD38 within EM CD8 T cells from symptomatic patients. The Spearman correlation test P value and the R coefficient are indicated in each graph. Data represent mean values±S.E.M. *P<0.05; **P<0.01; ***P<0.001; ****P<0.0001. Symptomatic patients (S, red), asymptomatic patients (AS, black) and healthy controls (C, white). (TIF) [file ppat.1009367.s001.tif]

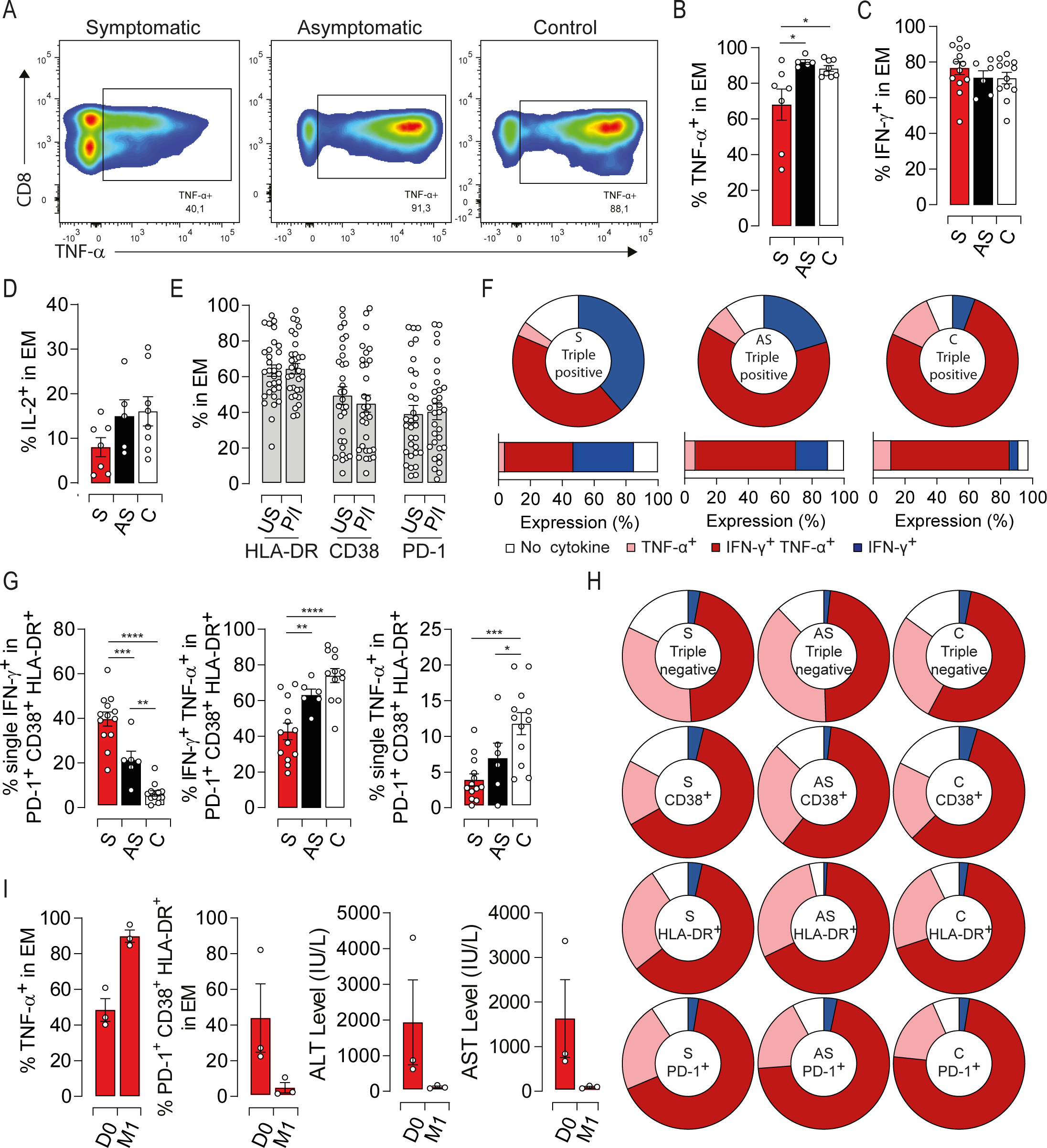

Supplement: S2 Fig — (A) Representative dot plots for TNF-α production in EM CD8 T cells. (B) Mean percentage of EM CD8 T cells producing TNF-α after overnight polyclonal stimulation (PMA/Ionomycin). (C and D) Bar graphs illustrating the frequency of EM CD8 T cells producing (C) IFN-γ or (D) IL-2. (E) Percentage of cells expressing HLA-DR, CD38 or PD-1 in unstimulated (US) or PMA/Ionomycin (P/I) stimulated EM CD8 T subset from all study group. (F) Donut charts representing the mean percentage of cells expressing different combination of IFN-γ and TNF-α in EM CD8 T cells identified by the triple expression (Triple positive) of PD-1, HLA-DR and CD38. (G) Bar graphs representing statistical analyses of donut chart from Triple positive cells. (H) Donut charts representing the mean percentage of cells expressing different combination of IFN-γ and TNF-α in EM CD8 T cells identified by the single expression of CD38, HLA-DR and PD-1 or the absence of these markers (Triple negative). (I) Comparison of indicated parameters in three symptomatic patients at the onset of symptoms (Day 0, D0) and at the convalescence period, one month later (M1). Data represent mean values±S.E.M. *P<0.05; **P<0.01; ***P<0.001; ****P<0.0001. Symptomatic patients (S, red), asymptomatic patients (AS, black) and healthy controls (C, white). (TIF) [file ppat.1009367.s002.tif]

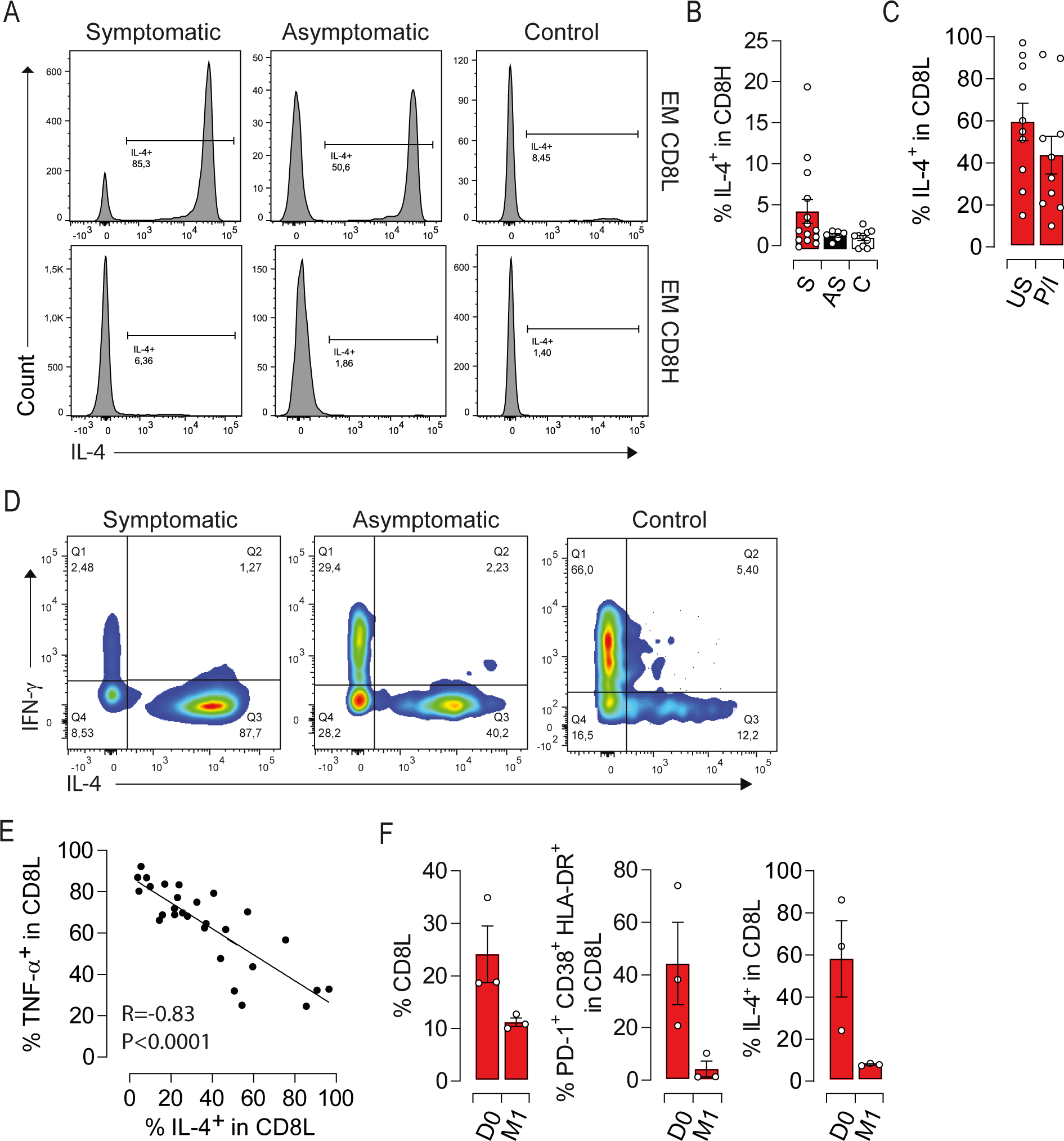

Supplement: S3 Fig — (A) Representative histograms of IL-4 production in CD8 EM cells expressing high (CD8H) or low (CD8L) levels of CD8. (B) Mean percentage of IL-4-producing cells in the CD8H subset. (C) Percentage of IL-4-producing cells in unstimulated (US) or PMA/Ionomycin (P/I) stimulated CD8L subset from symptomatic patients. (D) Representative dot plots of IFN-γ and IL-4 expression in CD8L cells. (E) Correlation between TNF-α and IL-4 productions in EM CD8L T cells by Spearman correlation test. The Spearman correlation test P value and R coefficient are indicated in the graph. (F) Comparison of indicated parameters in three symptomatic patients at the onset of symptoms (Day 0, D0) and at the convalescence period, one month later (M1). Data represent mean values ± S.E.M. Symptomatic patients (S, red), asymptomatic patients (AS, black) and healthy controls (C, white). (TIF) [file ppat.1009367.s003.tif]

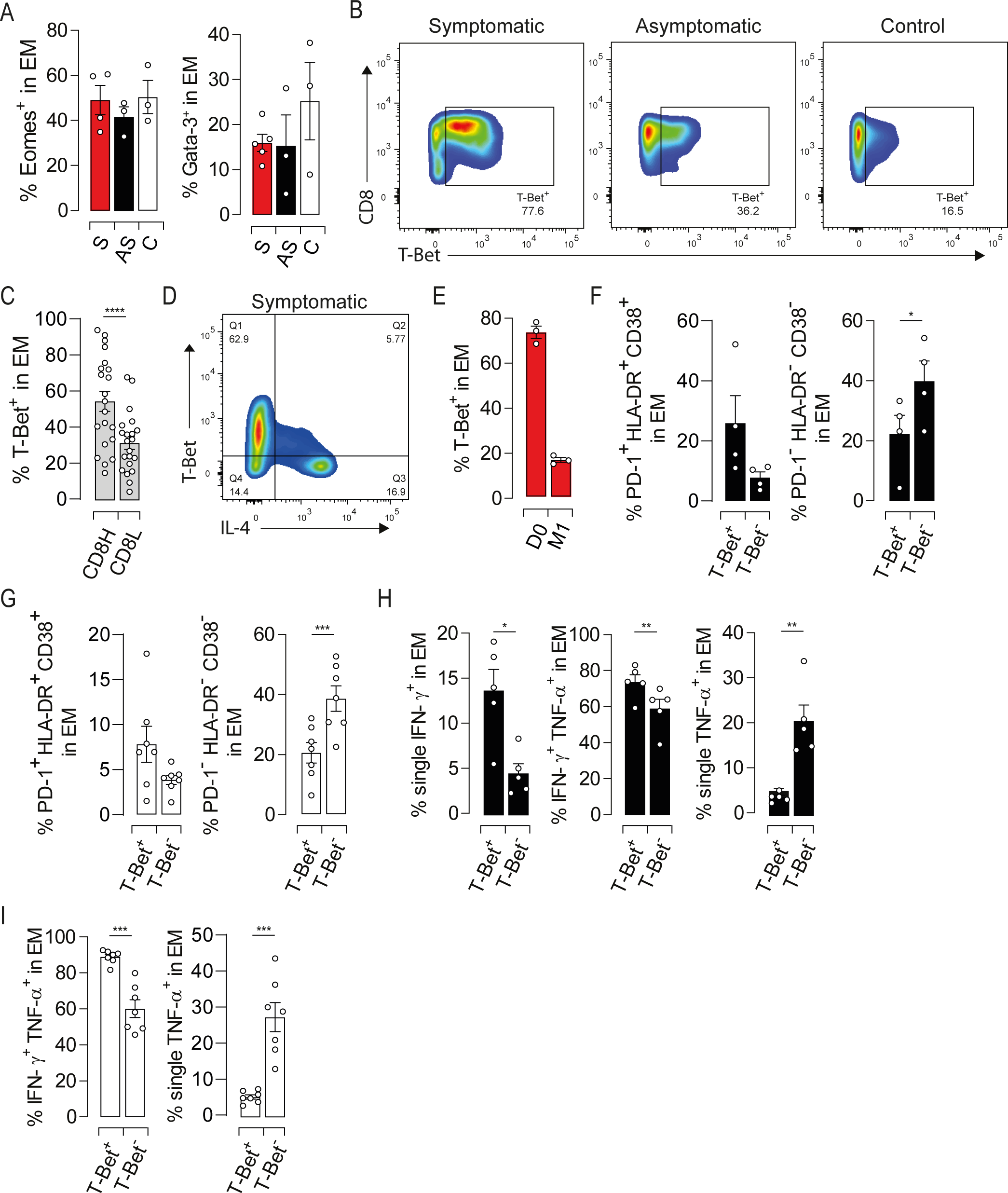

Supplement: S4 Fig — (A) Mean percentage of EM CD8 T cells expressing Eomes (left panel) and Gata-3 (right panel). (B) Representative dot plots of T-Bet expression in the EM compartment. (C) Mean percentage of T-Bet positive cells within the EM CD8 T cells expressing high (CD8H) or low (CD8L) levels of CD8 from all study group. (D) Representative dot plots of T-Bet and IL-4 expression in EM cells. (E) Comparison of T-bet expression in three symptomatic patients at the onset of symptoms (Day 0, D0) and at the convalescence period, one month later (M1). (F and G) Frequency of cells identified by triple expression (left panel) or absence (right panel) of PD-1, HLA-DR and CD38 in the T-Bet positive and negative subset from AS patients (F) and Controls (G). (H and I) Bar graphs representing the mean percentage of cells from AS patients (H) and Controls (I) identified by single/double expression patterns of IFN-γ and TNF-α. Bar graphs represent statistical analyses of the donut charts (right panel). Data represent mean values±S.E.M. *P<0.05; **P<0.01; ***P<0.001;****P<0.0001. Symptomatic patients (S, red), asymptomatic patients (AS, black) and healthy controls (C, white). (TIF) [file ppat.1009367.s004.tif]

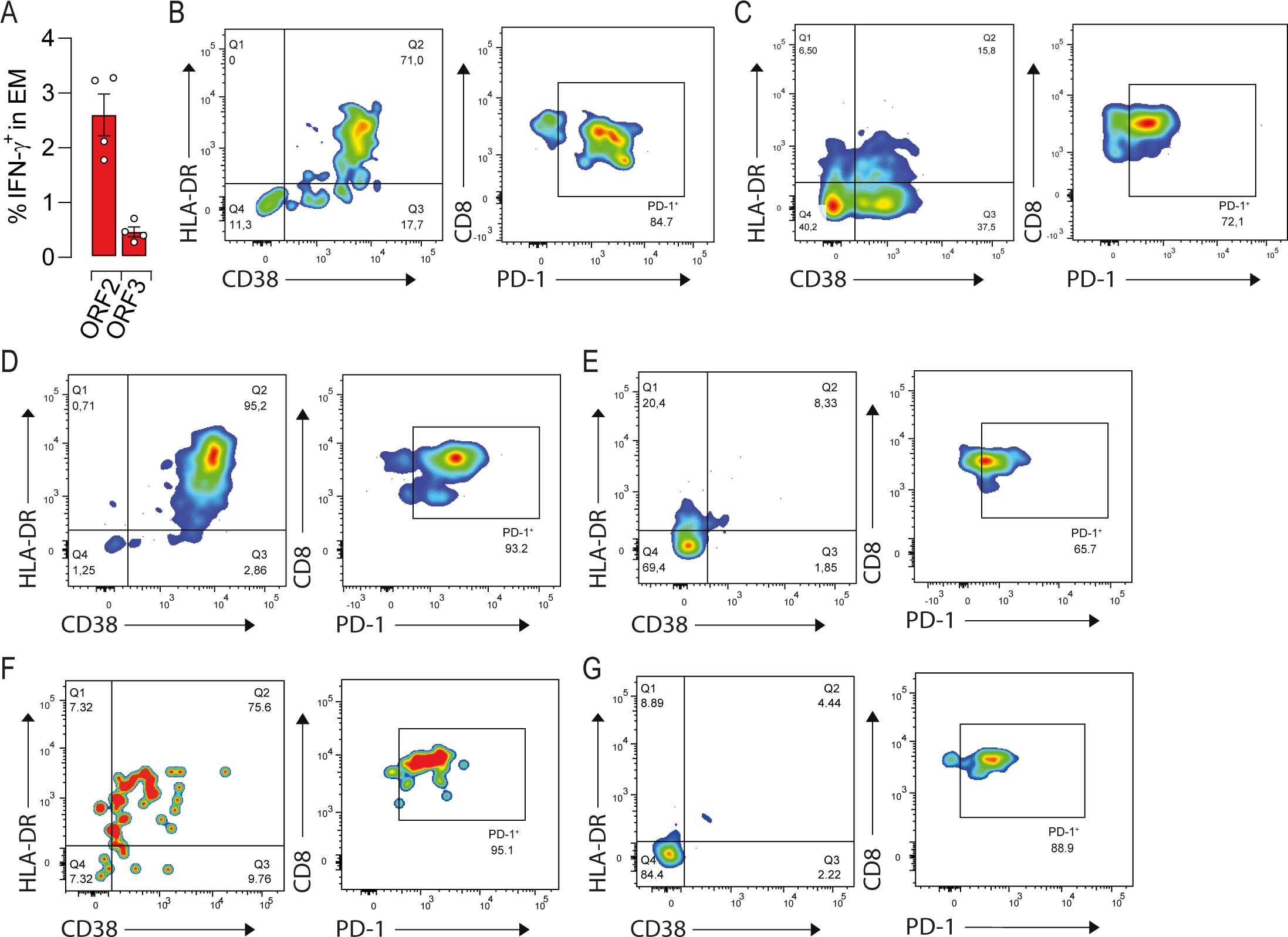

Supplement: S5 Fig — (A) Frequency of IFN-γ positive cells in EM following stimulation with ORF2 or ORF3. (B and C) Representative dot plots of HLA-DR/CD38 coexpression (left panel) and PD-1 expression (right panel) in HEV-3 specific EM CD8 T cells from symptomatic (B) and asymptomatic (C) patients upon stimulation with ORF2 peptides. (D and E) Representative dot plots of HLA-DR/CD38 (left panel) and PD-1 (right panel) expression in hCMV specific EM CD8 T cells from symptomatic patients (D) and Controls (E). (F and G) Representative dot plots of HLA-DR/CD38 (left panel) and PD-1 (right panel) expression in EBV specific EM CD8 T cells from symptomatic patients (F) and Controls (G). Symptomatic patients (S, red). (TIF) [file ppat.1009367.s005.tif]

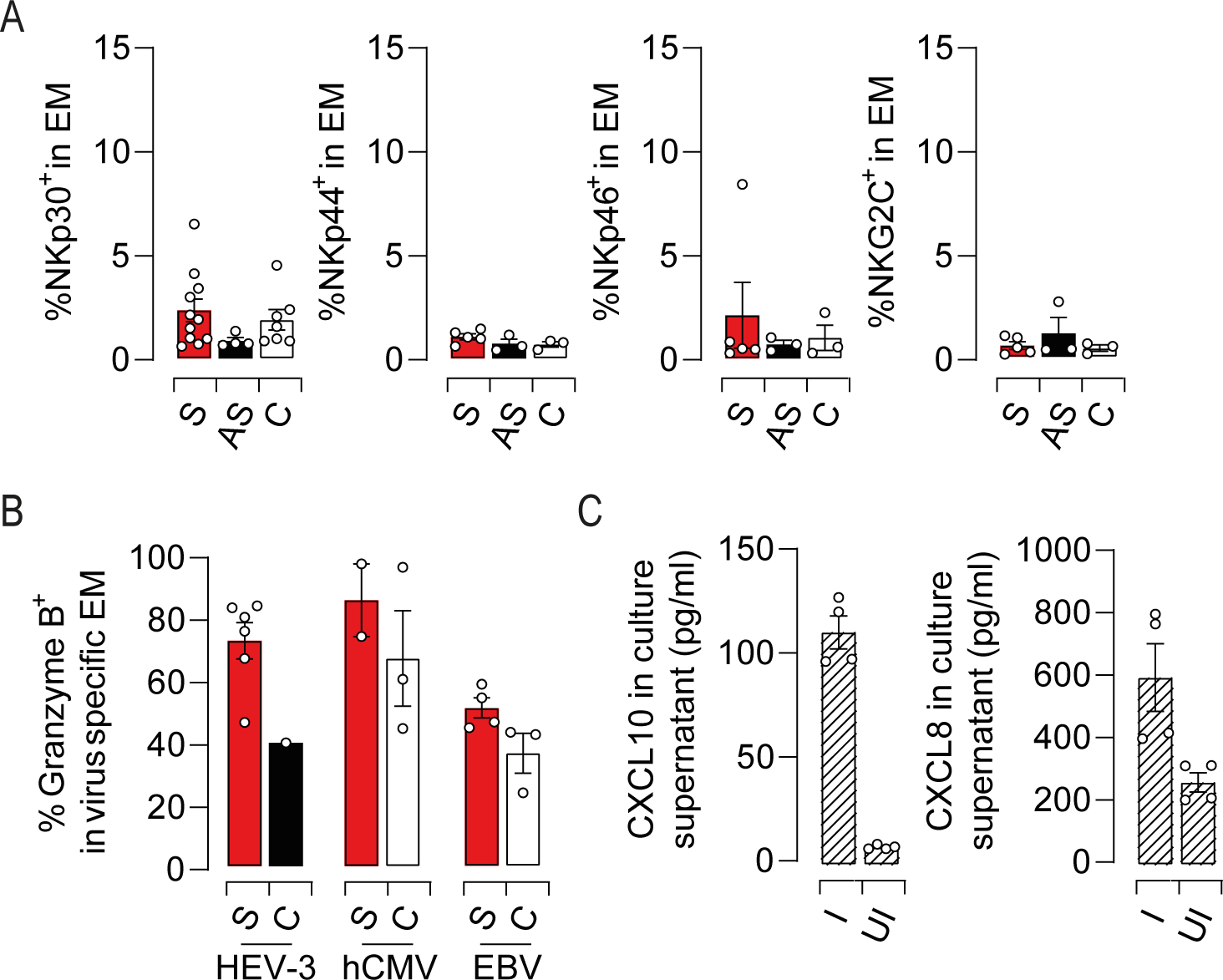

Supplement: S6 Fig — (A) Frequency of cells expressing the indicated activating Natural Killer receptors in the EM compartment. (B) Frequency of granzyme B positive cells in HEV-3-, hCMV- and EBV-specific EM CD8 T cells. (C) Chemokine levels in HepG2 cell culture supernatant (I, infected; UI, uninfected). Symptomatic patients (S, red), asymptomatic patients (AS, black) and healthy controls (C, white). (TIF) [file ppat.1009367.s006.tif]

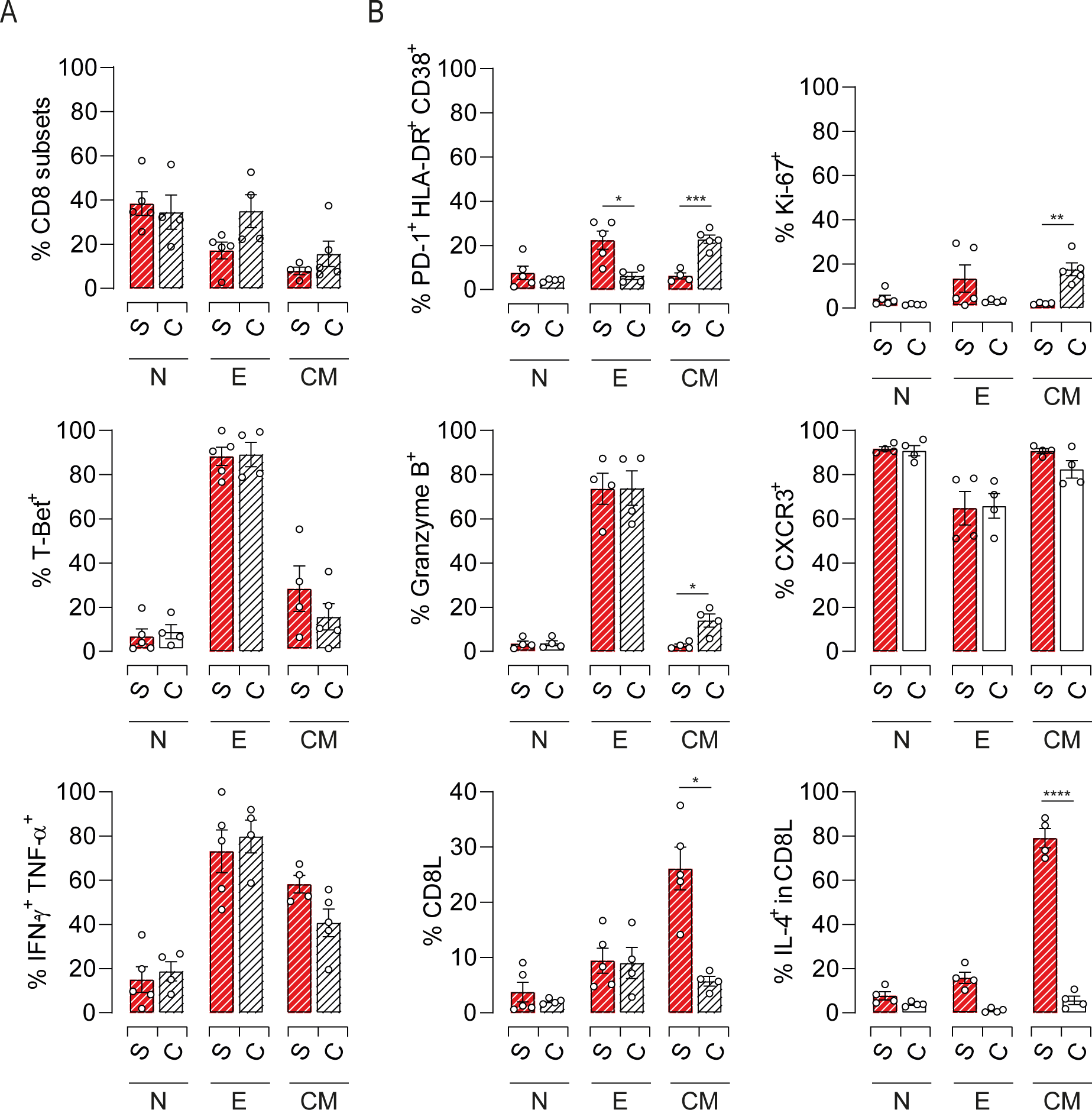

Supplement: S7 Fig — (A) Mean percentage of CD8 T subsets: naïve (N), effector (E), effector memory (EM) and central memory (CM). (B) Percentage of cells expressing indicated markers in CD8 T subsets. Data represent mean values±S.E.M. *P<0.05; **P<0.01; ***P<0.001; ****P<0.0001. Younger patients (S, red hatch pattern) and young healthy controls (C, white hatch pattern). (TIF) [file ppat.1009367.s007.tif]
