# Supplementary material for: Effector memory CD8 T cell response elicits Hepatitis E Virus genotype 3 pathogenesis in the elderly
Source: PLoS Pathog. 2021 Feb 22;17(2):e1009367. doi: 10.1371/journal.ppat.1009367 (PMC7932504; doi:10.1371/journal.ppat.1009367)
Supplement: Attachment — Submitted filename: Response to Reviewers.docx [file ppat.1009367.s008.docx]

**Author response to Reviewers' comments**

**Major Issues: Key Experiments Required for Acceptance**

Reviewer #1:

*1. The authors need to examine further the phenotype and functional characteristics of bystander-activated CD8 T cells. For example, T-bet expression, loss of TNF production, and increased production of IL-4 need to be examined in the gate of bystander-activated CD8 T cells (e.g. CMV-HLA-A2 pentamer+CD8+ T cells).* *Regarding bystander activation of memory CD8 T cells, please show additional examples other than CMV-HLA-A2 pentamer+CD8+ T cells (e.g. EBV-specific or flu-specific).*

We followed the reviewer suggestion and added the phenotypic and functional characteristics of EBV-specific EM cells using HLA-A2 pentamer refolded with BMLF-1epitopes. We further characterized the response of bystander-activated EM cells by assessing the expression of Ki-67, granzyme B, NKG2D, TNF-α, and IL-4 in EBV- and CMV-specific EM cells (Fig 6-7 and Fig S5-6). Our findings are in agreement with the global immune profile of EM cells from symptomatic patients, further strengthening our original hypothesis that both HEV-3-specific and -non-specific EM CD8 T cells are at play during symptomatic HEV-3 infection.

*2. In addition, the expression of NK receptors need to examined in HEV-3-specific CD8 T cells and bystander-activated CD8 T cells (e.g. CMV-HLA-A2 pentamer+CD8+ T cells), including NKG2D. It was previously shown that NKG2D is upregulated only in IL-15-induced bystander-activated CD8 T cells, not in TCR-stimulated CD8 T cells (Kim J et al. Immunity. 2018 Jan 16;48(1):161-173.e5. doi: 10.1016/j.immuni.2017.11.025.).*

We would like to thank the reviewer for this valuable suggestion. We examined the expression of the main Natural Killer activating receptors (NKp30, NKp44, NKp46, NKG2C and NKG2D) on EM cells. Except NKG2D, NKRs were barely detected in the EM compartment (Fig 7b and Fig 6Sa). Symptomatic patients harbored a considerable frequency of highly activated EM cells bearing NKG2D (Fig 7c). In addition, we found that the pro-inflammatory cytokines IL-15 and IL-18 were significantly increased in symptomatic patients compared to asymptomatic ones (Fig 7a). Together, these new data suggest that NKG2D might contribute to HEV-3 pathogenesis in IL-15-dependent manner.

*3. For functions of effector memory CD8 T cells, the authors focused on cytokine production and cytokine polyfunctionality. However, it is also required to investigate a cytotoxic capacity of effector memory CD8 T cells by staining perforin and granzymes.*

To unravel the cytotoxic capacity of EM cells we assessed the expression of granzyme B. We found that symptomatic patients exhibit high levels of granzyme B (Fig 5e). This expression is correlated with both activation status of EM and T-Bet expression (Fig 5f-g). In addition, the frequency of NKG2D and granzyme B double positive cells was strikingly elevated in symptomatic patients further strengthening the involvement of NKG2D in HEV-3 pathogenesis (Fig 7d-e and Fig S6b).

*Reviewer #2: The overall sample size of the studied cohort is very small (n=20), in particular, the symptomatic patients seem to be significantly older than the other two groups. The authors are therefore advised to include further subjects with similar age into their study. In particular, the samples of elderly asymptomatic patients and control patients. Furthermore, since the authors do not include longitudinal samples, it remains unclear, whether the loss of cytokine production (i.e. TNFalpha) is just occurs as a temporal phenomenon or also remains after the patients clear the virus.* *The authors only employ samples from older patients, however it seems relevant to compare the observed effects of HEV on the composition on immune cells to samples of younger patients to directly allow conclusions regarding age-related effects of an HEV infection.*

We followed the reviewer suggestion and enrolled additional age-matched subjects in the study as follows: nine symptomatic patients, one asymptomatic patient and seven controls. Consequently, the age gap between study groups is lower with no significant difference (Table 1). The inclusion of additional patients consolidated our previous data and conclusions.

We also included a cohort of young patients and matched-healthy control (Table 2). Accordingly, our data suggest that younger patients share with elderly ones some features of EM cell response but delineate differences that might explain why elderly patients are at high risk to develop severe forms of Hepatitis E. Indeed, we show now that aging is associated with increased inflammation, a shift toward Th2 profile and high cytotoxic potential (Fig 8 and Fig S7).

Activation threshold, T-Bet expression and cytokine production were assessed in three symptomatic patients one month after the onset of the symptoms. HLA-DR/CD38/PD-1 expression was drastically decreased compared to the early stage of the infection. In addition, TNF-α and IL-4 production, and T-Bet expression restored to healthy control levels (Fig S2i, S3f and S4e). The recovery of EM cell response coincided not only with viral clearance but also with a decline in the liver enzyme levels and resolution of symptoms (Fig S2i), strengthening the notion that EM cells are responding to HEV-3 infection.

*Reviewer #3: In general, this is an interesting and important research topic and the authors are (to their and my knowledge) the first to address the often severe pathogenesis of HEV-3 in the elderly. There are, however, several major limitations that need to be at least carefully discussed in a revised version:*

*1. The authors had the aim to determine the mechanisms underlying HEV-3 pathogenesis in the elderly. Is the pathogenesis of symptomatic HEV-3 in the elderly different from that in younger patients? Why did the authors not include a control group of young patients with acute-symptomatic HEV-3?*

We would like to thank the reviewer for this suggestion that was also raised by reviewer#2. We included a cohort of young patients and matched-healthy control (Table 2). Accordingly, our data suggest that younger patients share with elderly ones some features of EM cell response but delineate differences that might explain why elderly patients are at high risk to develop severe forms of Hepatitis E. Indeed, we show now that aging is associated with increased inflammation, shift toward Th2 profile and high cytotoxic potential (Fig 8 and Fig S7).

*2. Is the activation of effector memory cells in acute-symptomatic HEV-3 in the elderly different from other viral infections such as Flu? Absolute changes in global T cell phenotypes are different to interpret without comparison to better understood settings. In other words, is this just a normal reaction to an acute viral infection or is this unique to HEV-3?*

Bystander activation of CD8 memory T cells including cells specific to hCMV and EBV has been previously reported during acute viral infections, such as the primary Human Immunodeficiency Virus, acute Hepatitis A and B Viruses and Hantavirus (1-4). However, we report here that this bystander activation is associated with key features of early T cell exhaustion, including an overexpression of inhibitory receptors along with qualitative and quantitative defects in cytokine production. This profile is generally observed during the chronic phase of viral infections. Given that EM cell phenotype normalized following patient recovery, the exhaustion-like profile emphasizes a robust ongoing CD8 T cell activation during the acute phase of HEV-3 infection. This issue is now discussed in the revised manuscript, lines 342-357 and 380-392.

*3. HEV-3-specific CD8+ T cell were only estimated by using ORF2-derived overlapping peptides, so that ORF1- and ORF3-specific CD8+ T cell were not analyzed. This might lead to an important bias in the assessment of HEV3-specific versus bystander effects.*

Although HEV-specific T-cell responses are detectable against non-structural proteins encoded in ORF1, most responses are detected against regions of the genome that are relatively well conserved, with the majority located in ORF2 (5-8). Most of the experimental vaccines against hepatitis E are therefore based on this antigenic protein (9-11). In agreement with these studies, we identified weak frequency of HEV-3-specific cells following stimulation with ORF3 peptides (Fig S5a). Therefore, we focused our work on ORF2-specific cells. We agree with the reviewer that targeting these cells alone may underestimate the frequency of HEV-3-specific cells. However, even if we take into account the percentage of cells specific to ORF1 and ORF3, the global frequency of HEV-3-specific cells remains low and does not explain the drastic changes of the EM compartment in symptomatic patients. Therefore, our hypothesis that bystander activation contributes to the observed alteration within the EM compartment remains valid. This issue is now addressed in newly added Fig S5a and in the discussion, lines 404-414.

*4. CMV-specific CD8+ T cells were activated in acute-symptomatic HEV3. The authors interpret this as bystander effect. This could also be a result of heterologous immunity (compare Soon et al., Frontiers in Immunology 2019).*

We agree with the reviewer that cross-reactive T-cell responses between heterologous viruses or against an autoantigen have been suggested to be extensive (12, 13). However, this issue is unlikely at play during HEV-3 infections since we showed that not only EM_hCMV_ cells displayed functional alterations but also EM_EBV_ (newly added Fig 6f and Fig S5f). In addition, HEV-3 epitope peptides do not exhibit, to our knowledge, significant homology with amino acid sequence from these viruses. Moreover, our newly added data suggest that the pro-inflammatory cytokine IL-15 and IL-18 are key drivers in EM cell activation during HEV-3 infection (Fig 7). This notion is now added to the discussion, lines 342-362.

**Minor Issues: Editorial and Data Presentation Modifications**

*Reviewer #1: 1. For IL-4 ICS, it seems that PBMCs were cultured without ex vivo stimulation (US in Figure S4C). However, I cannot find a detailed method for this assay. It is important because ICS is usually performed with ex vivo stimulation.*

For cytokine detection, PBMC (10^6^ cells) were incubated for 3 hours or overnight with/without 50ng/ml phorbol-12-myristate-13-acetate and 1µM ionomycin (Sigma-Aldrich). DMEM:F-12 (V:V) medium supplemented with 10% fetal calf serum (FCS) was used for cell culture. To enable intracellular cytokine accumulation, GolgiPlug was added in stimulated and unstimulated conditions. Missing details are now added to the methods section. In contrast to type 1 cytokines (IFN-γ, TNF-α and IL-2), IL-4 was detected *ex vivo* without further stimulation. In addition, polyclonal stimulation did not induce further IL-4 production in HEV-3 patients (Fig S3c), strengthening the notion that IL-4 is induced *in vivo* under pathological conditions. Albeit unusual in healthy subjects, similar spontaneous cytokine production were previously described during acute illness (14).

*2. In Figure 2E, I understood that ‘% HLA-DR(+)’ means ‘% HLA-DR(+)CD38(-)PD-1(-)’. If this is the case, please clearly re-write the y-axis. (same for ‘% CD38+’ and ‘% PD-1+’).*

To make clear that single-positive cells are meant in Fig 2e, we now changed “ % HLA-DR+ in EM” to “% single HLA-DR+ in EM”. This issue is now corrected in Fig 2e and all the figures of the revised manuscript.

*Reviewer #2: The authors frequently use s.e.m. during their boxplots and might consider to display values of the individual patients throughout the manuscript.* *Its not clear how the cluster of patients were identified during the PCA analysis in Fig.1F.*

We followed the reviewer’s advice and changed all the figures accordingly. We used scatter graphs in which each plot represents the value obtained for single patient/control.

Principal Component Analysis was performed using ClustVis (http://biit.cs.ut.ee/clustvis/). Values were centered, unit variance scaling was applied to rows and single value decomposition with imputation was used to calculate principal components. Each plot represent the dimensionality reduction of the expression level of several markers by one subject. X and Y axis show PC1 and PC2 that explain 67.6% and 10.5% of the total variance, respectively. Patients were classified into three groups, Symptomatic, Asymptomatic or Control, and the software generated the prediction ellipse accordingly. Prediction ellipses are such that, with probability of 0.95, a new observation from the same group will fall inside the ellipse. The prediction ellipse of control group do not cross the ellipse of symptomatic group. By contrast, the prediction ellipse of control group is intermingled with the one of asymptomatic patients. This strengthen the conclusion that EM cells from symptomatic patients are highly activated whereas asymptomatic patients are endowed with only low-to-moderate EM cell response.

*Reviewer #3:*

*1. Table 1: 1/5 patients with “asymptomatic” course had fever. Did he have signs of a bacterial infection focus? Otherwise, he/she had a symptomatic course by definition?*

Unfortunately, no extensive research was carried out on this patient. Therefore, we cannot exclude a bacterial infection. Despite fever, the biochemical and immunological profiles of this patient were comparable to those from other asymptomatic patients. In addition, symptomatic patients were classified based on the liver specific symptoms including mucosal icterus, colored urine and discolored stools. Therefore, this issue should not affect our study.

*2. Table 1: Please state normal ranges especially for bilirubin (since the micromolar range is not that common).*

The normal levels of ALT and AST are below 45 UI/L for men and 35 for women, and bilirubin is below 17µM. This information has been added to the methods section.

*3. Figures, e.g. Fig. 2e: Please change “%HLA-DR+ in EM” to e.g. “% only HLA-DR+ in EM” to make clear that single-positive cells are meant here.*

To make clear that single-positive cells are meant in Fig 2e, we now changed “ % HLA-DR+ in EM” to “% single HLA-DR+” in EM. This issue is now corrected in Fig 2e and all the figures of the revised manuscript.

*4. Fig. 3b: light/dark blue (IFNy+TNF+ and IL2+, respectively) and light/dark purple (TNF+IL2+ and IFNy+IL2+, respectively) are difficult/impossible to distinguish.*

We thank the reviewer for alerting us that the colors of figure 3b (now Fig 3c) are hard to distinguish. To simplify the donut charts and make our message clearer, the polyfunctional cytokine production is now focused on IFN-γ and TNF-α and the figures were modified accordingly.

References

1.	Doisne JM, Urrutia A, Lacabaratz-Porret C, Goujard C, Meyer L, Chaix ML, et al. CD8+ T cells specific for EBV, cytomegalovirus, and influenza virus are activated during primary HIV infection. J Immunol. 2004;173(4):2410-8.

2.	Kim J, Chang DY, Lee HW, Lee H, Kim JH, Sung PS, et al. Innate-like Cytotoxic Function of Bystander-Activated CD8(+) T Cells Is Associated with Liver Injury in Acute Hepatitis A. Immunity. 2018;48(1):161-73 e5.

3.	Sandalova E, Laccabue D, Boni C, Tan AT, Fink K, Ooi EE, et al. Contribution of herpesvirus specific CD8 T cells to anti-viral T cell response in humans. PLoS Pathog. 2010;6(8):e1001051.

4.	Tuuminen T, Kekalainen E, Makela S, Ala-Houhala I, Ennis FA, Hedman K, et al. Human CD8+ T cell memory generation in Puumala hantavirus infection occurs after the acute phase and is associated with boosting of EBV-specific CD8+ memory T cells. J Immunol. 2007;179(3):1988-95.

5.	Al-Ayoubi J, Behrendt P, Bremer B, Suneetha PV, Gisa A, Rinker F, et al. Hepatitis E virus ORF 1 induces proliferative and functional T-cell responses in patients with ongoing and resolved hepatitis E. Liver Int. 2018;38(2):266-77.

6.	Bowen DG, Warren A, Davis T, Hoffmann MW, McCaughan GW, Fazekas de St Groth B, et al. Cytokine-dependent bystander hepatitis due to intrahepatic murine CD8 T-cell activation by bone marrow-derived cells. Gastroenterology. 2002;123(4):1252-64.

7.	Gisa A, Suneetha PV, Behrendt P, Pischke S, Bremer B, Falk CS, et al. Cross-genotype-specific T-cell responses in acute hepatitis E virus (HEV) infection. J Viral Hepat. 2016;23(4):305-15.

8.	Husain MM, Aggarwal R, Kumar D, Jameel S, Naik S. Effector T cells immune reactivity among patients with acute hepatitis E. J Viral Hepat. 2011;18(10):e603-8.

9.	Li SW, Zhang J, Li YM, Ou SH, Huang GY, He ZQ, et al. A bacterially expressed particulate hepatitis E vaccine: antigenicity, immunogenicity and protectivity on primates. Vaccine. 2005;23(22):2893-901.

10.	Shrestha MP, Scott RM, Joshi DM, Mammen MP, Jr., Thapa GB, Thapa N, et al. Safety and efficacy of a recombinant hepatitis E vaccine. N Engl J Med. 2007;356(9):895-903.

11.	Wu T, Wu XL, Ou SH, Lin CX, Cheng T, Li SW, et al. Difference of T cell and B cell activation in two homologous proteins with similar antigenicity but great distinct immunogenicity. Mol Immunol. 2007;44(12):3261-6.

12.	Soon CF, Zhang S, Suneetha PV, Antunes DA, Manns MP, Raha S, et al. Hepatitis E Virus (HEV)-Specific T Cell Receptor Cross-Recognition: Implications for Immunotherapy. Front Immunol. 2019;10:2076.

13.	Welsh RM, Selin LK. No one is naive: the significance of heterologous T-cell immunity. Nat Rev Immunol. 2002;2(6):417-26.

14.	Walker D, Jason J, Wallace K, Slaughter J, Whatley V, Han A, et al. Spontaneous cytokine production and its effect on induced production. Clin Diagn Lab Immunol. 2002;9(5):1049-56.
